# Supplementary material for: Identifying repeat domains in large genomes
Source: Genome Biol. 2006 Jan 31;7(1):R7. doi: 10.1186/gb-2006-7-1-r7 (PMC1431705; doi:10.1186/gb-2006-7-1-r7)
Supplement: Additional File 1 — A zipped file of browsable HTML files with a complete list of the connected components in the repeat domain graph of human Repbase. [file gb-2006-7-1-r7-S1.gz › html/subgraphs/8024.pdf]

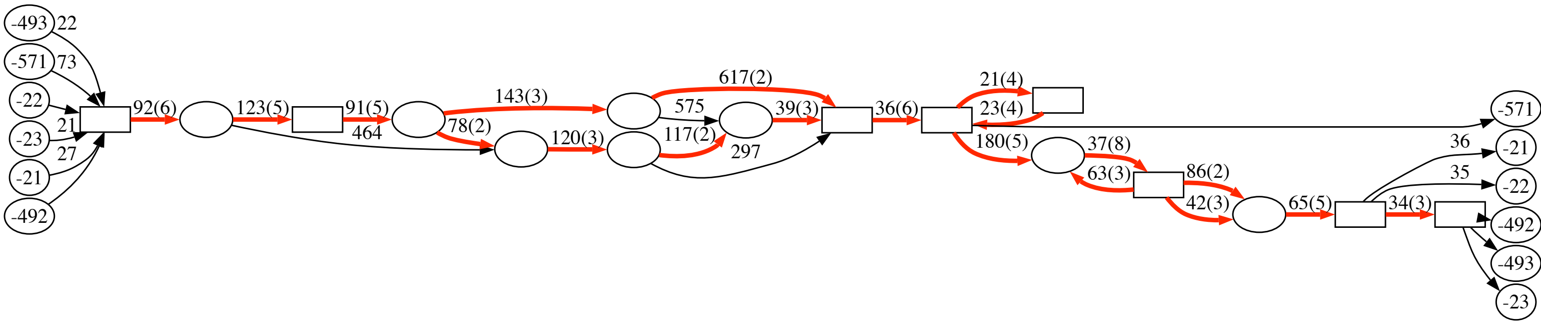

-493
22
-571
73
-22
-23
21
27
-21
-492
92(6)
123(5)
464
91(5)
143(3)
78(2)
120(3)
617(2)
575
117(2)
297
39(3)
36(6)
21(4)
23(4)
180(5)
37(8)
63(3)
86(2)
42(3)
65(5)
34(3)
36
35
-571
-21
-22
-492
-493
-23
